# Durability Benchmarking of Contemporary Second-Line and Later Therapies for Relapsed or Refractory Follicular Lymphoma Using Reconstructed Individual Patient Data From Published Kaplan-Meier Curves

**DOI:** 10.7759/cureus.111312

**Published:** 2026-06-22

**Authors:** Oscar J Burke, Nicolas Peruzzo, Nimra Tul Ain Khan, Pragnan Kancharla

**Affiliations:** 1 Internal Medicine, MedStar Union Memorial Hospital, Baltimore, USA; 2 Hematology and Medical Oncology, MedStar Franklin Square Medical Center, Baltimore, USA

**Keywords:** bispecific antibody, chimeric antigen receptor (car) t-cell therapy, follicular lymphoma, progression-free survival (pfs), relapsed lymphoma, restricted mean survival time

## Abstract

Background

Patients with relapsed or refractory (R/R) follicular lymphoma (FL) after two or more prior lines of therapy can now be treated with mechanistically distinct modalities, including CD19-directed chimeric antigen receptor (CAR) T-cell therapy, CD20×CD3 bispecific antibodies, and a Bruton tyrosine kinase inhibitor plus anti-CD20 combination. Head-to-head data are lacking, and sequencing decisions are made with limited comparative evidence. We benchmarked durability outcomes across pivotal trials using reconstructed individual patient data (rIPD) derived from published Kaplan-Meier (KM) curves.

Methods

KM curves and numbers-at-risk tables from pivotal prospective studies in ≥2-line R/R FL were digitized and rIPD-reconstructed using a validated algorithm: axicabtagene ciloleucel (ZUMA-5), tisagenlecleucel (ELARA), lisocabtagene maraleucel (TRANSCEND FL), mosunetuzumab, epcoritamab (EPCORE NHL-1), and zanubrutinib plus obinutuzumab versus obinutuzumab (ROSEWOOD). Prespecified durability endpoints were landmark progression-free survival (PFS) at 12 and 24 months and restricted mean survival time to 24 months (RMST24). Overall survival (OS) landmarks were summarized where follow-up permitted. ROSEWOOD served as an internal validity check using a Cox model fit to reconstructed data.

Results

At 24 months, landmark PFS was 65.4% (95% confidence interval (CI) 52.0-82.4) for liso-cel, 62.0% (50.2-76.6) for axi-cel, 53.8% (45.2-63.9) for zanubrutinib plus obinutuzumab, 49.3% (39.3-61.7) for mosunetuzumab, 43.7% (31.3-61.1) for epcoritamab, and 24.7% (14.9-40.9) for obinutuzumab monotherapy. Twelve-month PFS ranged from 81.8% (74.5-89.8) with liso-cel and 77.9% (69.4-87.5) with axi-cel to 61.6% (53.6-70.8), 60.4% (50.8-72.0), and 59.1% (50.5-69.1) for zanubrutinib plus obinutuzumab, mosunetuzumab, and epcoritamab, respectively; tisagenlecleucel showed 12-month PFS of 68.3% (57.2-81.6) with shorter follow-up (maximum 18.2 months). PFS RMST24 estimates were 19.5 months for liso-cel, 18.6 for axi-cel, 16.5 for zanubrutinib plus obinutuzumab, 16.2 for mosunetuzumab, 14.8 for epcoritamab, and 11.9 for obinutuzumab. OS at 24 months was high across regimens (mosunetuzumab 87.3%, liso-cel 84.6%, axi-cel 82.8%, zanubrutinib plus obinutuzumab 77.3%, and epcoritamab 67.6%). In ROSEWOOD, reconstructed data reproduced the published PFS benefit for zanubrutinib plus obinutuzumab (hazard ratio (HR) 0.48, 95% CI 0.32-0.71; p < 0.001) with a weaker OS signal (HR 0.61, 0.35-1.07; p = 0.08).

Conclusions

In this rIPD-based durability benchmark for ≥2-line R/R FL, the estimated landmark PFS and RMST24 values varied across regimens, with numerically higher point estimates for the CAR T-cell therapies and the zanubrutinib plus obinutuzumab combination and numerically lower estimates for the bispecific antibodies and the obinutuzumab control; CIs overlapped substantially, and 24-month OS estimates were broadly similar across regimens. Because the source trials enrolled materially different populations, these side-by-side estimates are descriptive benchmarks and should not be read as head-to-head comparisons of efficacy. Faithful internal reproduction of the randomized ROSEWOOD effect supports reconstruction fidelity. The analysis provides a transparent, durability-focused reference framework to support sequencing discussions and hypothesis generation, not comparative effectiveness conclusions.

## Introduction

Follicular lymphoma (FL) is the most common indolent non-Hodgkin lymphoma and follows a relapsing-remitting course in most patients. Although outcomes after first-line immunochemotherapy are generally favorable, the subset of patients who progress within 24 months of starting first-line therapy (POD24) experience markedly inferior survival, and outcomes deteriorate with each subsequent line of treatment [1,2].

For patients with relapsed or refractory (R/R) FL after two or more prior lines, the therapeutic landscape has expanded rapidly to include mechanistically distinct options. CD19-directed chimeric antigen receptor (CAR) T-cell products, axicabtagene ciloleucel (axi-cel), tisagenlecleucel (tisa-cel), and lisocabtagene maraleucel (liso-cel), deliver a single, fixed cellular treatment [3-7]. CD20×CD3 bispecific antibodies, mosunetuzumab and epcoritamab, offer off-the-shelf, time-limited, or continuous outpatient regimens [8-10]. The Bruton tyrosine kinase inhibitor zanubrutinib combined with the anti-CD20 antibody obinutuzumab provides a chemotherapy-free oral-plus-antibody combination [11]. Each was studied in single-arm trials (except ROSEWOOD, which randomized against obinutuzumab monotherapy), and no randomized head-to-head comparisons exist among them.

In the absence of comparative trials, clinicians and decision-makers rely on cross-trial observation to weigh options, yet published reports vary in the endpoints, summary statistics, and follow-up they present, complicating like-for-like comparison. Reconstructed individual patient data (rIPD) from published Kaplan-Meier (KM) curves and numbers-at-risk tables enable a common analytic framework in which standardized landmark estimates and restricted mean survival time (RMST) can be derived consistently across studies [12-14]. RMST, the area under the survival curve to a fixed horizon, summarizes durability without relying on the proportional-hazards assumption and is well-suited to cross-study description [14].

In current practice, sequencing decisions among these mechanistically distinct options must be made without head-to-head trials, and this analysis was designed to inform those discussions rather than to rank the therapies. We therefore performed a durability-focused benchmarking analysis of contemporary ≥2-line R/R FL therapies using rIPD. The primary objective was descriptive: to place standardized landmark progression-free survival (PFS), RMST, and overall survival (OS) estimates for these regimens on a common analytic footing so that their durability profiles can be viewed side by side. The secondary objective was methodological: to test the fidelity of the reconstruction by reproducing the randomized within-trial treatment effect reported in ROSEWOOD. This was explicitly not a comparative effectiveness study, and we did not aim to establish head-to-head superiority among regimens.

## Materials and methods

Study selection and data sources

We assembled a curated set of pivotal prospective trials of contemporary therapies for ≥2-line R/R FL (grade 1-3A) with published KM curves and accompanying numbers-at-risk tables: axi-cel (ZUMA-5) [3,4], tisa-cel (ELARA) [5,6], liso-cel (TRANSCEND FL) [7], mosunetuzumab [8,9], epcoritamab (EPCORE NHL-1 follicular cohort) [10], and zanubrutinib plus obinutuzumab versus obinutuzumab monotherapy (ROSEWOOD) [11]. For each regimen and endpoint, when more than one publication or data cut was available, we preferred the report with the longest follow-up that also provided a numbers-at-risk table and digitizable curves for that endpoint; primary-analysis curves were used when no suitable longer-follow-up update was available. This was a curated benchmarking exercise of pivotal trials rather than a formal systematic review, and no formal risk-of-bias scoring was performed. Only published, aggregate, de-identified data were used; no individual patient records were accessed, and institutional review board approval was therefore not required.

Digitization and IPD reconstruction

Published KM curves were digitized with WebPlotDigitizer version 5.2 (Ankit Rohatgi; https://automeris.io/WebPlotDigitizer), capturing closely spaced coordinate points along each curve after calibrating the plot axes to the published axis ranges. rIPD (time and event/censoring indicator) were reconstructed from the digitized coordinates and the reported numbers-at-risk using the algorithm of Guyot et al., implemented in the IPDfromKM R package [12,13]. Reconstructed survival times were constrained to be non-negative; small negative values arising from digitization rounding (all within approximately one month of zero) were set to zero, and survival functions were enforced to be monotonically non-increasing. Reconstructed sample sizes matched the reported analyzed cohorts. As further quality-control checks, the reconstruction was anchored to the reported numbers-at-risk at each published time point, and each reconstructed KM curve was checked against the source curve to confirm faithful reproduction; the recovery of the randomized ROSEWOOD hazard ratio (HR), reported below, provided an additional quantitative validation of the reconstruction pipeline.

Endpoints

The prespecified primary durability endpoints were landmark PFS at 24 months and PFS RMST to 24 months (RMST24); landmark PFS at 12 months was a key secondary endpoint. Additional endpoints were landmark OS at 12 and 24 months, PFS and OS RMST to 36 months where follow-up permitted, and, where reported, duration of response (DoR; responders only) and time to next treatment (TTNT). Endpoint definitions follow those of the source trials; PFS was based on the assessment (investigator or independent review) reported in each source publication, consistent with Lugano response criteria [15].

Statistical analysis

For each reconstructed dataset, the KM estimator was computed, and landmark survival probabilities with 95% confidence intervals (CIs) were obtained at 12, 24, and 36 months, using the complementary log-log transformation for interval estimation. RMST was estimated as the area under the KM curve up to each prespecified horizon (24 and 36 months) and was reported only when a regimen's maximum follow-up reached that horizon [14]. To provide descriptive cross-study summaries, landmark estimates were pooled within modality classes and overall using random-effects meta-analysis (restricted maximum likelihood) on the complementary log-log scale, with heterogeneity quantified by I²; pooled estimates are descriptive and are reported in the supplementary outputs rather than as comparative effect estimates. As an internal validity check, a Cox proportional-hazards model was fit to the pooled ROSEWOOD reconstructed arms to estimate the HR for zanubrutinib plus obinutuzumab versus obinutuzumab for PFS and OS, and the reconstructed HRs were compared with the published trial results [11]. Analyses were performed in R using the survival and metafor packages [16]. No formal cross-trial hypothesis testing between non-randomized regimens was performed. Throughout, regimen-level estimates are reported descriptively and side by side to benchmark durability; they are not statistically compared with one another, and ordinal language implying relative efficacy (for example, more or less “durable”) is intentionally avoided.

All data analyzed in this study were derived from published KM figures and numbers-at-risk tables in the cited trials. The reconstructed individual patient data, the digitized curve coordinates, and the full analysis code (digitization-to-reconstruction and all landmark, RMST, and Cox analyses in R) have been deposited in the Zenodo repository (https://doi.org/10.5281/zenodo.20764080); they also remain available from the corresponding author on request.

## Results

Included studies and follow-up

Seven treatment arms from six trials were analyzed, spanning three CAR T-cell products, two bispecific antibodies, and the randomized ROSEWOOD comparison (Table 1). Reconstructed cohorts ranged from 72 to 145 patients, and maximum reconstructed follow-up ranged from approximately 18 months (ELARA) to approximately 46 months (mosunetuzumab). Because the ELARA follow-up extended only to about 18 months, 24-month landmark and RMST24 estimates were not estimable for tisa-cel, and only 12-month landmarks are reported for that regimen.

**Table 1:** Included studies, modality, analyzed cohort size, maximum reconstructed follow-up, and primary data source. N denotes the reconstructed analyzed cohort. CAR: chimeric antigen receptor; BTKi: Bruton tyrosine kinase inhibitor; Ab: antibody; PFS: progression-free survival; OS: overall survival; NCT: ClinicalTrials.gov identifier; mo: months

Regimen	Modality	Trial (NCT)	N	Max follow-up, PFS/OS (mo)	Primary data source
Lisocabtagene maraleucel	CAR T-cell	TRANSCEND FL (NCT04245839)	101	24.5/24.3	Morschhauser et al., 2024 [7]
Axicabtagene ciloleucel	CAR T-cell	ZUMA-5 (NCT03105336)	86	25.8/37.3	Jacobson et al., 2022; Neelapu et al., 2024 [3,4]
Tisagenlecleucel	CAR T-cell	ELARA (NCT03568461)	94	18.2/18.3	Fowler et al., 2022 [5]
Mosunetuzumab	Bispecific Ab	GO29781 (NCT02500407)	90	37.3/46.2	Budde et al., 2022; Sehn et al., 2025 [8,9]
Epcoritamab	Bispecific Ab	EPCORE NHL-1 (NCT03625037)	128	28.4/30.1	Linton et al., 2024 [10]
Zanubrutinib + obinutuzumab	BTKi + anti-CD20	ROSEWOOD (NCT03332017)	145	42.7/44.7	Zinzani et al., 2023 [11]
Obinutuzumab (control)	Anti-CD20	ROSEWOOD (NCT03332017)	72	38.2/44.3	Zinzani et al., 2023 [11]

Baseline patient and disease characteristics across trials

The included cohorts differed substantially in baseline patient and disease characteristics, as summarized in Table 2 (values are abstracted from the source publications for the relevant cohort and data cut, not derived from the reconstruction). Although all enrolled patients with ≥2-line R/R grade 1-3A FL, the trials varied in median age (57 to 66 years), the median number of prior lines (3 to 4), the proportion with POD24 (34% to 63%), the proportion refractory to the last prior line (32% to 78%), prior autologous stem-cell transplantation (19% to 36%), and performance-status eligibility (Eastern Cooperative Oncology Group (ECOG) 0-1 for the CAR T-cell and mosunetuzumab trials versus 0-2 for epcoritamab and ROSEWOOD). Refractoriness was also defined and reported inconsistently: a discrete double-refractory rate (refractory to both an anti-CD20 antibody and an alkylator) was reported as 68% in ELARA, 64% in TRANSCEND FL (3L+), 53% in mosunetuzumab, and 70% in epcoritamab, whereas ZUMA-5 and ROSEWOOD did not report a discrete double-refractory percentage. Critically, the analysis populations also differ in kind: the CAR T-cell estimates derive from infused or efficacy-evaluable patients (that is, those who completed leukapheresis, any bridging, and manufacturing), whereas the bispecific cohorts reflect all enrolled and treated patients, and ROSEWOOD reflects randomized (intention-to-treat) arms. TRANSCEND FL additionally enrolled a separate second-line high-risk cohort that was not part of the ≥3-line population benchmarked here.

**Table 2:** Baseline patient and disease characteristics across the included trials, abstracted from the source publications. Values are abstracted from the cited source publications [3-5,7-11] to characterize between-trial heterogeneity and were not derived from the reconstruction. POD24 denotes progression within 24 months of starting first-line therapy as reported by each trial; for TRANSCEND FL, the value shown is POD24 from initiation of first-line anti-CD20 plus alkylator therapy (POD24 from diagnosis was 43%). Refractory to last line denotes no response to, or progression during or within approximately 6 months of, the most recent prior therapy (definitions varied slightly across trials). Prior ASCT for epcoritamab is from the EPCORE NHL-1 follicular-cohort congress report; for ROSEWOOD, it is the overall trial value (arms not reported separately). EE: efficacy-evaluable; ITT: intention-to-treat; ASCT: autologous stem-cell transplantation; ECOG: Eastern Cooperative Oncology Group performance-status eligibility; FL: follicular lymphoma; 3L+: third-line and later; liso-cel: lisocabtagene maraleucel; axi-cel: axicabtagene ciloleucel; tisa-cel: tisagenlecleucel; ZO: zanubrutinib plus obinutuzumab; O: obinutuzumab

Regimen (population)	Median age (y) (range)	Prior lines, median (range)	POD24 (%)	Refractory to last line (%)	Prior ASCT (%)	Stage III-IV (%)	Analysis set; ECOG
Liso-cel (TRANSCEND FL, 3L+)	62 (23-80)	3 (2-10)	54	64	31	88	Infused/EE; 0-1
Axi-cel (ZUMA-5, FL)	60 (53-67)	3 (2-4)	55	68	24	85	Infused/EE; 0-1
Tisa-cel (ELARA, FL)	57 (49-64)	4 (2-13)	63	78	36	86	Infused/EE; 0-1
Mosunetuzumab	60 (29-90)	3 (2-10)	52	69	31	77	Enrolled/treated; 0-1
Epcoritamab	65 (55-72)	3 (2-4)	42	69	19	85	Enrolled/treated; 0-2
ZO (ROSEWOOD)	63 (31-84)	3 (2-11)	34	32	21	82	ITT (randomized); 0-2
O (ROSEWOOD)	66 (32-88)	3 (2-9)	42	40	21	83	ITT (randomized); 0-2

Landmark PFS

**Figure 1:**
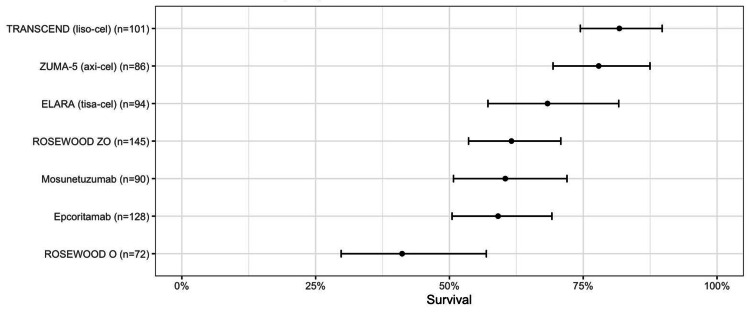
Landmark progression-free survival at 12 months across regimens, estimated from reconstructed individual patient data. Points are the Kaplan-Meier estimates of progression-free survival at 12 months, and horizontal bars are the 95% confidence intervals; n denotes the number of patients in the reconstructed analyzed cohort. Axi-cel: axicabtagene ciloleucel; liso-cel: lisocabtagene maraleucel; O: obinutuzumab; tisa-cel: tisagenlecleucel; ZO: zanubrutinib plus obinutuzumab

**Figure 2:**
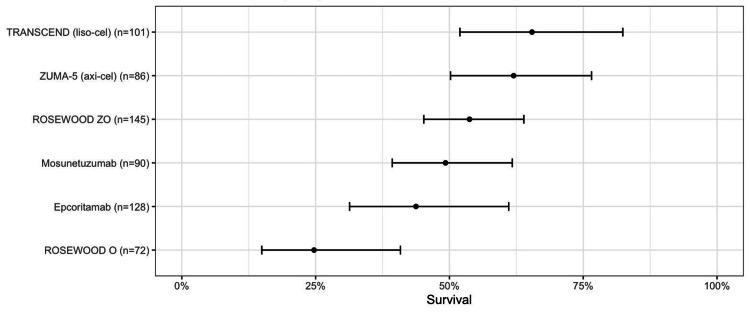
Landmark progression-free survival at 24 months across regimens, estimated from reconstructed individual patient data. Points are the Kaplan-Meier estimates of progression-free survival at 24 months, and horizontal bars are the 95% confidence intervals; n denotes the number of patients in the reconstructed analyzed cohort. Tisagenlecleucel (ELARA) is not shown because follow-up did not reach 24 months. Axi-cel: axicabtagene ciloleucel; liso-cel: lisocabtagene maraleucel; O: obinutuzumab; ZO: zanubrutinib plus obinutuzumab

Reconstructed landmark PFS estimates are summarized in Table 3 and Figures 1, 2; because they derive from separate trials in non-identical populations (Table 2), they are presented as within-regimen descriptive estimates rather than comparisons. At 12 months, estimated PFS was 81.8% (95% CI 74.5-89.8) with liso-cel, 77.9% (69.4-87.5) with axi-cel, 68.3% (57.2-81.6) with tisa-cel, 61.6% (53.6-70.8) with zanubrutinib plus obinutuzumab, 60.4% (50.8-72.0) with mosunetuzumab, and 59.1% (50.5-69.1) with epcoritamab; the obinutuzumab monotherapy control arm was 41.2% (29.8-56.9). At 24 months, estimated PFS was 65.4% (52.0-82.4) with liso-cel, 62.0% (50.2-76.6) with axi-cel, 53.8% (45.2-63.9) with zanubrutinib plus obinutuzumab, 49.3% (39.3-61.7) with mosunetuzumab, and 43.7% (31.3-61.1) with epcoritamab, with the obinutuzumab control at 24.7% (14.9-40.9); tisa-cel was not estimable at 24 months. The CIs were wide and overlapped extensively across the active regimens, and the values were not statistically compared.

**Table 3:** Landmark progression-free survival (PFS) at 12, 24, and 36 months (mo) from reconstructed individual patient data. NE: not estimable (maximum follow-up did not reach the landmark time); CI: confidence interval

Regimen	PFS at 12 mo (%) (95% CI)	PFS at 24 mo (%) (95% CI)	PFS at 36 mo (%) (95% CI)
Lisocabtagene maraleucel	81.8 (74.5-89.8)	65.4 (52.0-82.4)	NE
Axicabtagene ciloleucel	77.9 (69.4-87.5)	62.0 (50.2-76.6)	NE
Tisagenlecleucel	68.3 (57.2-81.6)	NE	NE
Zanubrutinib + obinutuzumab	61.6 (53.6-70.8)	53.8 (45.2-63.9)	43.4 (32.2-58.6)
Mosunetuzumab	60.4 (50.8-72.0)	49.3 (39.3-61.7)	42.5 (31.9-56.6)
Epcoritamab	59.1 (50.5-69.1)	43.7 (31.3-61.1)	NE
Obinutuzumab (control)	41.2 (29.8-56.9)	24.7 (14.9-40.9)	13.5 (5.6-32.5)

Restricted mean survival time

Estimated RMST values are shown in Table 4 and represent the area under each reconstructed curve to the specified horizon. PFS RMST24 was 19.5 months with liso-cel, 18.6 months with axi-cel, 16.5 months with zanubrutinib plus obinutuzumab, 16.2 months with mosunetuzumab, 14.8 months with epcoritamab, and 11.9 months with the obinutuzumab control. Where follow-up reached 36 months, PFS RMST36 was 22.1 months with zanubrutinib plus obinutuzumab, 21.7 months with mosunetuzumab, and 14.0 months with the obinutuzumab control. OS RMST24 estimates were similar across regimens (range approximately 19.4-22.5 months). As with the landmark estimates, these values are descriptive and were not statistically compared across regimens.

**Table 4:** Restricted mean survival time (mo) for progression-free survival (PFS) and overall survival (OS) to 24- and 36-mo horizons. RMST: restricted mean survival time; NE: not estimable (maximum follow-up did not reach the horizon, or endpoint not analyzed); mo: months

Regimen	PFS RMST24 (mo)	PFS RMST36 (mo)	OS RMST24 (mo)	OS RMST36 (mo)
Lisocabtagene maraleucel	19.5	NE	22.0	NE
Axicabtagene ciloleucel	18.6	NE	22.3	31.7
Tisagenlecleucel	NE	NE	NE	NE
Zanubrutinib + obinutuzumab	16.5	22.1	21.4	30.3
Mosunetuzumab	16.2	21.7	22.5	32.8
Epcoritamab	14.8	NE	19.4	NE
Obinutuzumab (control)	11.9	14.0	19.8	27.5

Landmark OS

**Figure 3:**
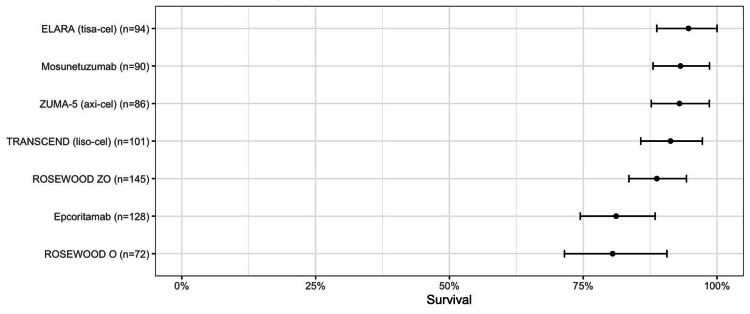
Landmark overall survival at 12 months across regimens, estimated from reconstructed individual patient data. Points are the Kaplan-Meier estimates of overall survival at 12 months, and horizontal bars are the 95% confidence intervals; n denotes the number of patients in the reconstructed analyzed cohort. Axi-cel: axicabtagene ciloleucel; liso-cel: lisocabtagene maraleucel; O: obinutuzumab; tisa-cel: tisagenlecleucel; ZO: zanubrutinib plus obinutuzumab

**Figure 4:**
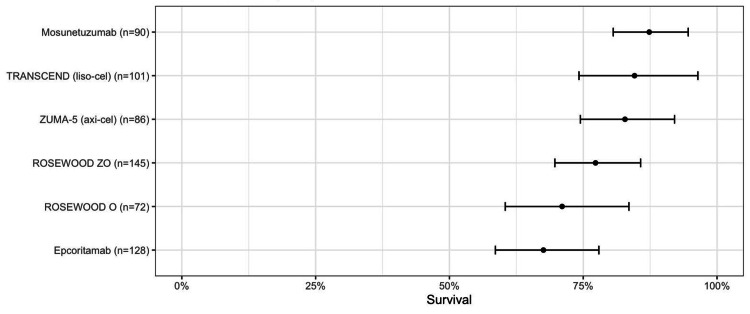
Landmark overall survival at 24 months across regimens, estimated from reconstructed individual patient data. Points are the Kaplan-Meier estimates of overall survival at 24 months, and horizontal bars are the 95% confidence intervals; n denotes the number of patients in the reconstructed analyzed cohort. Tisagenlecleucel (ELARA) is not shown because follow-up did not reach 24 months. Axi-cel: axicabtagene ciloleucel; liso-cel: lisocabtagene maraleucel; O: obinutuzumab; ZO: zanubrutinib plus obinutuzumab

Estimated landmark OS values are summarized in Table 5 and Figures 3, 4, and show less spread than the PFS estimates. At 12 months, estimated OS exceeded 80% for every regimen (94.7% (88.7-100.0) with tisa-cel, 93.2% (88.1-98.6) with mosunetuzumab, 93.0% (87.7-98.6) with axi-cel, 91.3% (85.7-97.3) with liso-cel, 88.7% (83.5-94.3) with zanubrutinib plus obinutuzumab, 81.1% (74.5-88.4) with epcoritamab, and 80.5% (71.5-90.6) with the obinutuzumab control). At 24 months, estimated OS was 87.3% (80.6-94.6) with mosunetuzumab, 84.6% (74.2-96.4) with liso-cel, 82.8% (74.5-92.1) with axi-cel, 77.3% (69.7-85.7) with zanubrutinib plus obinutuzumab, 71.1% (60.4-83.5) with the obinutuzumab control, and 67.6% (58.6-77.9) with epcoritamab; tisa-cel was not estimable at 24 months. These estimates are descriptive and were not statistically compared.

**Table 5:** Landmark overall survival (OS) at 12, 24, and 36 mo from reconstructed individual patient data. NE: not estimable (maximum follow-up did not reach the landmark time); CI: confidence interval; mo: months

Regimen	OS at 12 mo (%) (95% CI)	OS at 24 mo (%) (95% CI)	OS at 36 mo (%) (95% CI)
Tisagenlecleucel	94.7 (88.7-100.0)	NE	NE
Mosunetuzumab	93.2 (88.1-98.6)	87.3 (80.6-94.6)	82.4 (74.3-91.5)
Axicabtagene ciloleucel	93.0 (87.7-98.6)	82.8 (74.5-92.1)	70.5 (55.0-90.3)
Lisocabtagene maraleucel	91.3 (85.7-97.3)	84.6 (74.2-96.4)	NE
Zanubrutinib + obinutuzumab	88.7 (83.5-94.3)	77.3 (69.7-85.7)	71.4 (62.1-82.1)
Epcoritamab	81.1 (74.5-88.4)	67.6 (58.6-77.9)	NE
Obinutuzumab (control)	80.5 (71.5-90.6)	71.1 (60.4-83.5)	49.1 (32.3-74.7)

DoR, TTNT, and ROSEWOOD internal validation

Among responders, the estimated 24-month DoR was 69.3% (59.8-80.2) with zanubrutinib plus obinutuzumab, 66.8% (55.5-80.3) with axi-cel, and 34.8% (19.6-62.0) with the obinutuzumab control; these responder-conditioned estimates come from different trials and are presented descriptively. In ROSEWOOD specifically, the one randomized comparison in this set, reconstructed 24-month TTNT was 68.4% (60.2-77.7) for zanubrutinib plus obinutuzumab versus 26.7% (17.0-41.9) for obinutuzumab monotherapy. Fitting a Cox model to the reconstructed ROSEWOOD arms reproduced the direction and magnitude of the published within-trial randomized effect (Table 6): the reconstructed PFS HR for zanubrutinib plus obinutuzumab versus obinutuzumab was 0.48 (95% CI 0.32-0.71; p < 0.001), closely matching the published estimate, with a weaker, non-significant OS signal (HR 0.61, 0.35-1.07; p = 0.08). Because this within-trial comparison can be benchmarked against a known randomized result, its concordance supports the fidelity of the reconstruction pipeline.

**Table 6:** Reconstructed Cox proportional-hazards treatment effect for zanubrutinib plus obinutuzumab (ZO) versus obinutuzumab (O) monotherapy in ROSEWOOD (internal validity check; n = 217). HRs estimated from reconstructed pooled arms; an HR below 1 favors the combination. HR: hazard ratio; CI: confidence interval

Endpoint	HR (ZO vs. O)	95% CI	p-value
Progression-free survival	0.48	0.32-0.71	<0.001
Overall survival	0.61	0.35-1.07	0.08

Safety and administration context from the source trials

Durability is only one dimension of treatment selection, and the modalities benchmarked here differ markedly in toxicity, administration, and logistics. Because these features cannot be derived from reconstructed survival curves, Table 7 summarizes safety and administration data as reported in the source publications, to be read alongside, not as an output of, the durability analysis. In broad terms, the CD19 CAR T-cell products are single, fixed infusions but are associated with cytokine release syndrome (CRS) and, distinctively, with immune effector cell-associated neurotoxicity syndrome (ICANS), graded by American Society for Transplantation and Cellular Therapy (ASTCT) consensus criteria [17], with axi-cel showing the highest grade ≥3 neurotoxicity among them and tisa-cel and liso-cel showing low grade ≥3 CRS/ICANS; CAR T-cell therapy also requires leukapheresis, a manufacturing interval (often with bridging therapy), delivery at certified centers, and a period of intensive monitoring. The CD20×CD3 bispecific antibodies are largely outpatient regimens using step-up dosing to mitigate CRS, which is common but predominantly low grade and concentrated in cycle 1, with low ICANS rates; mosunetuzumab is fixed-duration (stopping at eight cycles with a complete response), whereas epcoritamab is administered continuously until progression. Zanubrutinib plus obinutuzumab is a chemotherapy-free oral-plus-antibody combination without CRS or ICANS, with cytopenias, infections, and infrequent atrial fibrillation or hemorrhage as the principal concerns, given continuously until progression. Treatment-related hospitalization, infection risk, immunoglobulin and B-cell recovery, quality of life, and cost/access differ accordingly; formal quality-of-life and health-economic comparisons were outside the scope of this analysis and are not reported here.

**Table 7:** Safety and administration profile of the included regimens, abstracted from the source publications (for context; not derived from the reconstruction). Safety values are from the cited source publications [3-5,7-11]; because data cuts and grading conventions differ across trials, they are not directly comparable. CRS and ICANS/neuro entries are any-grade/grade ≥3 percentages; for tisagenlecleucel, the neurologic-event rate is shown with the ICANS-specific rate in parentheses. “Low” indicates mosunetuzumab's reported neurotoxicity was uncommon and predominantly low grade. Axi-cel rates are for the ZUMA-5 safety population (FL and marginal zone lymphoma). The obinutuzumab monotherapy arm (ROSEWOOD) had no CRS or ICANS and lower myelosuppression than the combination. CRS: cytokine release syndrome (ASTCT-graded where applicable); ICANS: immune effector cell-associated neurotoxicity syndrome; SC: subcutaneous; IV: intravenous; AF: atrial fibrillation; liso-cel: lisocabtagene maraleucel; axi-cel: axicabtagene ciloleucel; tisa-cel: tisagenlecleucel; ZO: zanubrutinib plus obinutuzumab

Regimen	CRS, any/≥3 (%)	ICANS/neuro, any/≥3 (%)	Setting	Duration	Principal non-CRS concerns
Axi-cel	82/7	59/19	Inpatient-capable; single infusion	One-time	Cytopenias, infections; manufacturing/bridging
Tisa-cel	49/0	37/3 (ICANS 4/1)	18% outpatient; single infusion	One-time	Cytopenias, infections; manufacturing/bridging
Liso-cel	58/1	15/2	13% outpatient; single infusion	One-time	Cytopenias, infections; manufacturing/bridging
Mosunetuzumab	44/2	Low	Outpatient; step-up dosing	Fixed (8-17 cycles)	Infections, neutropenia; injection-site reactions (SC)
Epcoritamab	67/2	6/0	Outpatient; SC, step-up dosing	Continuous to progression	Infections (incl. COVID-19), injection-site reactions
ZO (ROSEWOOD)	None	None	Outpatient; oral + IV antibody	Continuous to progression	Thrombocytopenia/neutropenia, diarrhea; rare AF/bleeding

## Discussion

Applying a single, consistent analytic framework to rIPD, we generated standardized landmark PFS, RMST, and OS estimates for contemporary ≥2-line R/R FL regimens and placed them side by side. The estimated PFS values spanned a wide range, with numerically higher point estimates for the CAR T-cell products and the zanubrutinib plus obinutuzumab combination and numerically lower estimates for the bispecific antibodies and the obinutuzumab control, whereas the estimated 24-month OS was high and similar across regimens, consistent with the indolent biology of FL, the availability of effective subsequent therapies, and immature OS follow-up for several regimens. We deliberately frame these as descriptive benchmarks rather than comparisons: the CIs overlapped extensively, no statistical testing between regimens was performed, and, as detailed below, the source trials enrolled materially different populations, so the ordering of point estimates cannot be attributed to the therapies themselves.

The agreement between our reconstructed ROSEWOOD treatment effect and the published randomized result is an important internal control. Recovering a PFS HR of 0.48 from digitized curves, closely matching the trial's reported benefit, indicates that the digitization and reconstruction pipeline preserved the underlying time-to-event structure and supports the credibility of the derived landmark and RMST estimates for the single-arm regimens.

These descriptive observations are consistent with the individual narratives of the source trials. Importantly, the differences in point estimates across regimens should not be read as evidence that any one regimen is more effective than another, because they may reflect not only the therapies but also substantial differences in the populations enrolled and analyzed. The comparison that can be interpreted causally is the within-trial randomized contrast in ROSEWOOD, which our reconstruction reproduced faithfully; cross-regimen differences require the cautious, non-comparative interpretation set out below.

Comparison with previous studies

Our descriptive findings are broadly concordant with prior cross-trial and indirect-comparison analyses in this setting, although our approach differs. Real-world series have shown that patients receiving third-line or later therapy for R/R FL, most often chemoimmunotherapy, have limited and progressively shorter responses and poorer survival with each successive line [18], consistent with the low landmark estimates we reconstructed for the obinutuzumab monotherapy control. Several groups have used population-adjustment methods to contextualize the newer agents against such benchmarks. A propensity-score-weighted comparison of ZUMA-5 against the international SCHOLAR-5 real-world cohort reported higher response rates, longer TTNT, and improved progression-free and OS for axi-cel relative to standard of care [19]. A matching-adjusted indirect comparison of the two earlier CAR T-cell products found broadly similar efficacy for tisagenlecleucel and axicabtagene ciloleucel after adjustment, with a more favorable safety profile for tisagenlecleucel [20], and a matching-adjusted comparison of lisocabtagene maraleucel with mosunetuzumab reported higher response and complete-response rates and improved PFS for the CAR T-cell product [21]. The direction of these adjusted comparisons, with CAR T-cell therapy outperforming both conventional real-world treatment and the mosunetuzumab bispecific, and the two CAR T-cell products performing broadly similarly to one another, parallels the ordering of our unadjusted point estimates.

Two differences from those analyses are important for interpretation. First, each of those comparisons formally adjusted for measured baseline differences between populations using matching or propensity weighting, whereas the present benchmark is deliberately descriptive and unadjusted and instead makes the between-trial differences explicit (Table 2); it is therefore best viewed as complementary to, rather than a substitute for, formal indirect comparison. Second, even after adjustment, several of those comparisons were limited by wide CIs or immature OS, which reinforces our central point that side-by-side estimates of these regimens should be read as hypothesis-generating rather than as evidence of differential efficacy.

Cross-trial heterogeneity

The central caveat for this benchmark is that the included trials, although all enrolling ≥2-line R/R grade 1-3A FL, differed across nearly every prognostically relevant axis (Table 2), and these differences plausibly account for much of the apparent separation in estimates. The number of prior therapies varied (median 3 to 4), as did the proportion of patients with POD24, a validated marker of poor prognosis: reported from the start of first-line therapy, POD24 affected 63% of ELARA, 55% of ZUMA-5, 54% of TRANSCEND FL (3L+), and 52% of mosunetuzumab patients, but only 42% of epcoritamab and 34% of ROSEWOOD (ZO) patients. Refractoriness likewise differed. The proportion refractory to the last prior line ranged from 64% to 78% across the single-arm CAR T-cell and bispecific trials but was only 32% in the ROSEWOOD combination arm, indicating a less treatment-refractory population in the randomized study. A discrete double-refractory rate (to an anti-CD20 antibody and an alkylator) was reported as 68% (ELARA), 64% (TRANSCEND FL 3L+), 53% (mosunetuzumab), and 70% (epcoritamab), but was not reported as a separate category in ZUMA-5 or ROSEWOOD, so this high-risk feature cannot be compared uniformly. Other high-risk markers were also defined inconsistently (for example, Follicular Lymphoma International Prognostic Index (FLIPI) high-risk ranged from 44% in ZUMA-5 to 61% in epcoritamab where reported, and bulky-disease and Groupe d'Etude des Lymphomes Folliculaires (GELF) or modified GELF thresholds differed), precluding direct comparison. Prior autologous stem-cell transplantation ranged from 19% (epcoritamab) to 36% (ELARA). Eligibility and fitness requirements were not uniform: the CAR T-cell trials and mosunetuzumab generally required ECOG performance status 0-1, whereas epcoritamab and ROSEWOOD permitted ECOG 0-2, potentially admitting less fit patients.

Beyond case mix, the analysis populations differ in kind, which is arguably the most important source of non-comparability. The CAR T-cell estimates are conditioned on patients who were successfully leukapheresed, bridged as needed, manufactured, and infused, and are frequently reported in efficacy-evaluable subsets; this introduces selection for patients fit and stable enough to reach infusion. In contrast, the bispecific antibody estimates reflect all enrolled and treated patients, and the ROSEWOOD estimates reflect randomized intention-to-treat arms, neither of which excludes patients lost during a manufacturing interval. A cross-trial juxtaposition therefore compares fundamentally different denominators. In addition, TRANSCEND FL uniquely enrolled a separate second-line high-risk cohort (all with POD24 from diagnosis and/or meeting modified GELF criteria); only the ≥3-line cohort (n = 101) was benchmarked here, but the trial's design and enrolled spectrum still differ from the others. For all of these reasons, the side-by-side estimates should be interpreted as descriptive context, and the construction of a comprehensive baseline-characteristics table (Table 2) is intended to make these differences explicit rather than to enable adjustment.

Other limitations

Several additional limitations apply. All regimens except ROSEWOOD were evaluated in single-arm trials without a shared comparator, so no causal or head-to-head inference is warranted. Reconstruction from published curves is an approximation whose accuracy depends on digitization quality and on the availability and granularity of numbers-at-risk tables; it operates at the level of the aggregate KM curve and cannot recover patient-level covariates for adjustment. Follow-up duration, data cutoffs, censoring conventions, and the use of investigator versus independent review for PFS differed across sources; landmark and RMST horizons were truncated at common time points to mitigate this, but residual differences remain, and tisa-cel (ELARA) could not be evaluated at 24 months because its available curves matured only to approximately 18 months. Duration-of-response estimates are conditional on achieving a response and are not directly comparable across regimens. The safety and administration data in Table 7 were abstracted from heterogeneous source reports using differing grading conventions and are provided only for context. Finally, this was a curated benchmark of pivotal trials rather than a formal systematic review, and the class-level pooled estimates are descriptive only.

Strengths and future directions

Strengths include the uniform analytic treatment of all curves, the use of RMST as a horizon-based durability measure that does not assume proportional hazards, the transparent presentation of between-trial heterogeneity, and explicit internal validation against a randomized trial. Together, these features make the analysis a transparent, reproducible reference summary of currently available durability data. Future work should incorporate population-adjusted indirect comparison methods, such as matching-adjusted indirect comparison, to account for measured between-trial differences in prognostic factors [22], integrate toxicity, quality-of-life, and health-economic dimensions for genuinely multidimensional treatment selection, and be updated as longer follow-up (including a more mature ELARA data cut) and additional randomized or real-world data become available.

## Conclusions

In this rIPD analysis, durability outcomes for contemporary therapies for second-line and later R/R FL were placed on a single, consistent analytic footing across trials that otherwise differ in design, follow-up, and reporting. Because all but one of the source trials were single-arm and the enrolled populations differed materially in prognostic characteristics and in how their analysis cohorts were defined, the resulting side-by-side estimates are descriptive benchmarks and should not be interpreted as head-to-head comparisons of efficacy. Faithful internal reproduction of the randomized within-trial effect from ROSEWOOD supports the fidelity of the reconstruction approach.

Interpreted within these limits, the analysis offers a transparent, reproducible, durability-focused reference framework that is most useful when considered alongside toxicity, administration, and access, and that can help frame treatment-sequencing discussions and generate hypotheses. Prospective comparative trials and population-adjusted analyses will be needed before firm comparative conclusions can be drawn.
